# Modulation of the post-auricular reflex in response to social and CT-optimal touch

**DOI:** 10.1371/journal.pone.0329625

**Published:** 2025-08-13

**Authors:** Birgit Hasenack, Anouk Keizer, Olga Wódecka, Leonard B. Schaafsma, H. Chris Dijkerman, David Terburg

**Affiliations:** 1 Faculty of Social and Behavioural Sciences, Experimental Psychology, Utrecht University, Utrecht, The Netherlands; 2 Faculty of Social and Behavioural Sciences, Clinical Psychology, Utrecht University, Utrecht, The Netherlands; 3 Department of Psychiatry and Mental Health, University of Cape Town, Cape Town, South Africa; University of Giessen: Justus-Liebig-Universitat Giessen, GERMANY

## Abstract

The pleasantness perception of CT-optimal touch is usually assessed with subjective and explicit measures. As these can be prone to biases, it is important to develop implicit measures as well. The vestigial post-auricular muscle reflex (PAR) might be a good candidate, given its sensitivity to pleasant visual and auditory stimuli. As such, we investigated if the PAR can also be modulated by CT-optimal touch. We additionally compared how the PAR responds to social and robotic touch and conducted control experiments to replicate the reflex’s specific sensitivity to primary rewards. The sample consisted of 43 non-clinical participants. PAR responses were recorded while participants were touched by an experimenter and a robot, with a velocity of 3 cm/s (CT-optimal touch) and 18 cm/s (CT non-optimal touch). After each trial, participants also subjectively rated the pleasantness of the touch. Although the results revealed that CT-optimal touch was subjectively perceived to be more pleasant than CT non-optimal touch, it did not result in a potentiation of the PAR. Interestingly, social touch was subjectively perceived to be more pleasant than robotic touch and potentiated the PAR. The control experiments confirmed that the PAR is particularly modulated by primary (food, erotica), and not secondary (adventure, cuteness, monetary) rewards. While additional research is needed to further investigate the relation between the PAR and CT-optimal touch, the current results do already suggest that this reflex responds to the primary reward value of social touch.

## 1. Introduction

Social touch is fundamental to human interaction and general wellbeing. It has been associated with the formation of social bonds [1] and can reduce pain [2,3], stress [4] and anxiety [5]. In the past two decades, an increasing number of studies have aimed to understand how this type of touch is perceived and processed. These studies have primarily focused on the role of low-threshold mechanoreceptor C-tactile (CT) fibers. CT fibers are exclusively found in hairy skin [6] and project to the posterior insular cortex [7,8]. These fibers respond most strongly to slow (1–10 cm) and gentle touch [6]. Touch applied at this speed is therefore also referred to as CT-optimal touch. Previous studies have repeatedly shown that CT-optimal touch is generally perceived as more pleasant than faster (CT non-optimal) touch [9].

Our current understanding of the pleasantness perception of CT-optimal touch is, however, almost exclusively based on subjective and explicit measures. The hedonic value of touch is usually assessed with Likert scales or visual analogue scales (VAS) [9]. Although assessing the subjective experience of touch is useful, these measures can also be prone to response biases. These can include central tendency (i.e., the tendency to select the middle or neutral option) [10], acquiescent responding (i.e., the tendency to respond positively to items) [11] and end-aversion (i.e., reluctance to select the most extreme options) [12]. The most problematic potential bias is perhaps the social desirability bias. This refers to the tendency to respond in a way that is perceived to be socially desirable [11]. In social touch studies, the researcher who administers the touch usually remains in the room while participants fill out the subjective measures. In some studies, participants are even asked to verbally rate the pleasantness of the touch (see, e.g., [13,14]). This could put additional pressure on participants to rate the touch as more pleasant than they actually perceived it to be, as they could be concerned that rating the touch as unpleasant will offend the researcher. Objective and implicit measures could circumvent the aforementioned biases, and subsequently provide important insights into the perception of CT-optimal touch. A few studies have already used such measures, including psychophysiological recordings of cardiac deceleration, heart rate variability, and activity in the zygomaticus major (for an overview, see [15]). However, as these measures are not widely implemented, we argue that it remains interesting to further investigate how pleasantness perception can be implicitly assessed.

A potential target for the development of an implicit measure of pleasantness perception is the post-auricular reflex (PAR). This is a response of the vestigial post-auricular muscle, which pulls the ears up and backwards [16]. The PAR can be evoked by short noise probes [17] and has been found to be modulated by emotional stimuli. Specifically, Benning et al. (2004) found that the magnitude of the PAR was potentiated when participants viewed pleasant images compared to unpleasant or neutral images [18]. This finding has since been replicated (see, e.g., [19,20]). The PAR has also been found to be potentiated by pleasant videos [21], facial expressions [22] and sounds [23]. Potentiation is therefore not exclusively associated with visual stimuli as it can be induced by other sensory modalities as well. Based on these results, the PAR has been proposed to reflect positive emotional [24] and appetitive motivational [20] processes.

An explanation for the emotional modulation of the PAR is provided by the nursing hypothesis. Johnson and colleagues (2012) based this hypothesis on the observation that baby mammals with moveable ears fold them back during nursing, thereby involving the postauricular muscle [25]. Through evolutionary processes, activity in this muscle might have subsequently become associated with other nursing-related stimuli (e.g., food or breasts). Although the PAR is now vestigial in humans, Johnson and colleagues (2012) argue that this association could have been preserved in mammals who are no longer able to move their ears. In support of their hypothesis, they found that potentiation of the PAR was stronger in response to appetitive stimuli when participants simultaneously mimicked a suckling motion by pursing their lips, compared to when they were smiling. The nursing hypothesis is also compatible with a study by Sandt and colleagues (2009). They found that potentiation of the PAR was strongest for primary rewards, such as images of food and erotica, with the latter including images of breasts [20]. This further supports the notion that the PAR is especially modulated by primary rewards that are related to the nursing process.

We could subsequently argue that the PAR can also be modulated by CT-optimal touch. Interpersonal touch plays an important role in the nursing process. Breastfeeding mothers have reported to affectionately stroke their babies while feeding them [26]. Interestingly, mothers have also been found to automatically stroke their infants at a CT-optimal speed [27]. Taken together, these findings indirectly suggest CT-optimal touch may be involved in the nursing process. The nursing hypothesis would then predict that CT-optimal touch would have become associated with the PAR through evolutionary processes, similar to what has already been observed for other nursing-related stimuli [20]. It should be noted that this association is not expected to have been developed for non-social forms of CT-optimal touch (e.g., touch administered by a robotic device). Although this is comparable to human-driven touch with respect to subjective pleasantness (see, e.g., [28,29]), nursing remains a deeply interpersonal process. Modulation of the PAR is therefore only expected to occur when CT-optimal touch is administered by another human being. Although this has not yet been investigated, previous studies have already found that the reflex can be modulated by nurturant pictures (see, e.g., [20,23,30]). These included images of social touch, such as a parent hugging a child [20]. In light of these findings, it does not seem unreasonable to assume that the PAR can also be modulated by (nurturant) social touch. However, it is important to further investigate this and assess if the potential modulation is specific for CT-optimal touch. This information is necessary to determine if the PAR can indeed be used to implicitly assess the pleasantness perception of CT-optimal touch.

The aim of the current study is therefore to investigate for the first time if social and CT-optimal touch can potentiate the PAR. Based on previous findings, we hypothesized that potentiation of the reflex would be stronger for CT-optimal touch than CT non-optimal touch. We additionally hypothesized that this modulation would be strongest when CT-optimal touch was administered by another human, and that social touch would generally result in a stronger potentiation than non-social touch. In all analyses, we controlled for the potential influence of longing for touch (LFT). LFT refers to a subjective discrepancy between the amount of touch one desires and receives [31]. It has already been shown that LFT can be positively associated with the subjective appetitive value of CT-optimal and CT non-optimal touch [32]. Since the PAR has been argued to be associated with the appetitive motivational system [20], we wanted to assess if potentiation could be similarly affected by LFT. Based on Meijer et al. (2022), we expected that LFT would be positively associated with the subjective pleasantness perception of CT-optimal and CT non-optimal touch, and with modulation of the PAR [32]. Lastly, we conducted a series of control experiments to validate our PAR measurements and to confirm that the reflex specifically responds to primary appetitive reward stimuli.

## 2. Method

### 2.1. Participants

Data were collected between 31-01-2024 and 30-06-2024. For the sample size, we aimed to include at least the same number of participants (*n* = 38) as Benning (2011), who showed reliable PAR modulation by positive compared to neutral and negative imagery (also see S1 File for our control experiment, which is a replication of the study by Benning) [23]. We continued collecting data after this sample was recruited, as we wanted to account for the potential removal of participants due to the physiological inclusion criteria (see *physiological recordings and preprocessing*). Recruitment of these additional participants continued for as long as time constraints allowed. Participants had to be older than 18 and not been diagnosed with a (self-reported) psychological, neurological or skin-related disorder. A total of 56 people participated in the study. After excluding data that did not meet the physiological inclusion criteria (see *physiological recordings and preprocessing*), 43 participants were included in the final analysis. The average age of this sample was 23.00 (*SD* = 3.15). Most participants were from the Netherlands (n = 33; 76.7%) and identified as women (n = 30; 69.8%).

### 2.2. Touch stimuli

**Fig 1:**
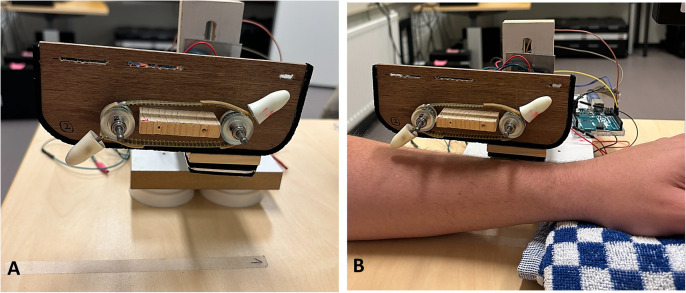
Robotic device. The robotic device consisted of two foam probes attached to a rotating band (A). A mark on the table (A) and a folded cloth (B) were used to correctly position the arm of the participant.

Two areas (9x4 cm) were marked on the top of the participant’s left forearm. These areas were alternately touched during the experiment to avoid habituation. The area that was touched first was randomized between participants. Touch was either administered at a CT-optimal (3 cm/s) or CT non-optimal (18 cm/s) speed. One of two (female and male) trained researchers applied social touch, while a robotic device (see Fig 1) was used to administer non-social touch. The robotic device was designed by Askari et al. (2021) and consisted of two foam probes (2 x 0.5 cm) that were attached to a v-belt [33]. This belt was rotated by a Nema 17 stepper motor (12V). The ensuing tactile sensation was designed to mimic the feeling of a fingertip stroking across the skin. In order to make the social and non-social touch as similar as possible, the researchers used their right index finger to administer touch. To ensure further consistency in the presentation of the tactile stimuli, the researchers extensively practiced with the application of social touch. They used video recordings of the robotic touch as a guideline for these practice sessions. During the experiment, they could see an 8-second countdown, which provided temporal guidance. The researchers additionally counted the number of times their finger had to move across the marked area during a trial (2 times for CT-optimal touch and 14 times between CT non-optimal touch). These numbers were not solely based on the duration of the trial, as we also had to take into consideration that the robotic device took minor breaks between the application of touches (1 second for CT-optimal touch and approximately 0.07 seconds for CT non-optimal touch). During the experiment, the researchers only touched participants with the same gender identification.

Participants used a computer-mouse to indicate how pleasant they perceived the touch on a scale ranging from “*Very unpleasant”* to “*Very pleasant”* in 1000 increments (pixels), thus resembling a VAS implemented in E-Prime 2.0 (see *Procedure*). At the end of the experiment, participants were also asked to rate how similar they perceived the social and robot touch on a VAS ranging from 0 (“*Not similar at all”*) to 100 (“*Very similar”*). This VAS was incorporated in Qualtrics (see *Procedure*). The average similarity rating was 40.21 (*SD* = 21.31).

### 2.3. Longing for touch

The LFT VAS was used to assess levels of LFT. This measure has been used in previous studies [32,34,35]. It consists of two items, on which participants indicate if they want to be touched more (or less) and if they want to touch others more (or less). The VAS ranges from 0 (“*Less”*) to 100 (“*More”*). The internal consistency of the scale was good in the current study, Cronbach’s α = .846. The average LFT score in the current sample was 57.95 (*SD* = 16.07).

### 2.4. Physiological recordings and preprocessing

EMG data was recorded with ActiView 8.06 software (BioSemi B.V.). A BioSemi Active-two system was used and the sampling rate was 2kHz. One electrode was placed on the postauricular muscle behind each ear, with a reference electrode placed on the posterior of each pinna [36]. The two BioSemi ground electrodes (CMS/DRL) were placed on the center of the forehead below the hairline. The PAR is evoked by short noise probes [17]. In the current experiment, these probes consisted of noise clicks (80 dB) that were presented at a rate of 10 Hz with a jitter of 10 ms. A total of 60 clicks were presented per trial. Clicks were presented using in-ear tubes connected to a sound delivery system attached to the upper-back of the participant to avoid artifacts in the EMG signal. The click signals were simultaneously relayed to an in-house developed BioSemi triggerbox [37], which sent a parallel-port signal upon reception allowing for precise synchronization of the EMG data with audio presentation.

The EMG data was preprocessed in Brain Vision Analyzer 2.1 (Brain Products GmbH). The recordings for each ear were kept separate in this process and the subsequent analysis. Raw data was first passed through band-pass (low: 8 Hz, high: 500 Hz) and notch (50 Hz) filters. The data was referenced (muscle- minus reference-signal) and segmented into epochs from 50 ms before each click to 100 ms after. The first 50 ms were subsequently checked for artifacts (fluctuations > 100 µV/ms or an overall difference > 150 µV). The epochs for each participant, ear and task-condition were averaged and baseline-corrected. In addition, a signal-to-noise ratio (SNR) was calculated for each ear using the full time-range (−50–100 ms) of the averages from the baseline trials using the built-in SNR function of Brain Vision Analyzer. As such, there were two baseline SNRs for each participant. All SNRs were inspected before the analyses were conducted. Data needed to have a baseline SNR above .01 to be included in the analyses, as values below this cut-off were judged to be insufficient to reliably score the PAR peaks. If the baseline SNR was below.01, all data for the associated ear was removed from the dataset. Nine participants had to be completely removed based on this criterion, and for twelve others only the data from one ear could be used in the subsequent analyses. Three additional participants had to be excluded due to technical errors that occurred during the experiment and one participant was removed for not filling out the LFT VAS. This meant that 43 participants were included in the final analyses. A baseline-to-peak measure was computed to calculate the PAR. Similar to Benning (2011), the peak was defined as the maximum EMG activity between 10 and 30 ms after the click [23]. The resulting PARs were log-transformed (natural logarithm).

### 2.5. Procedure

The experiments were built in E-Prime 2.0 (Psychology Software Tools, 2012). Qualtrics (Qualtrics, Provo, UT) was used to collect demographic information and the post-experiment questionnaires. All participants provided digital informed consent at the start of the experiment. They were then seated behind a table and in front of a computer. On the table, two marks were placed. These were used to ensure that all participants were similarly positioned with respect to the researcher and robot, and that touch was always applied to the same locations on the forearm. Where necessary, a folded cloth was used to adjust the distance between the participant’s arm and the robotic device. Participants first answered questions about their gender, age and nationality. Before starting with the main experiment, participants also received a test trial of each touch condition (social CT-optimal touch, social CT non-optimal touch, robot CT-optimal touch and robot CT non-optimal touch) to familiarize them with the procedure. These trials were also used to check if the participant’s arm was positioned correctly with respect to the robotic device. The researcher specifically assessed if the tip of the foam probes touched the arm of the participant without bending. After these test trials, participants were given the in-ear earphones and instructed to look at the computer screen for the remainder of the experiment.

**Fig 2:**
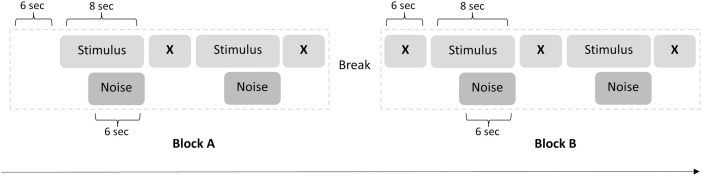
Visualization of the experimental procedure. A visualization of two blocks is represented in the figure, with the experiment consisting of 24 blocks in total. The same touch stimulus was presented in trials of a corresponding block. X represents the fixation cross that was presented on a computer screen. The duration of the break varied slightly between participants.

A visualization of the main experimental procedure can be found in Fig 2. During each trial, a fixation cross was shown on the computer screen. On a second screen, that was hidden from the participants, the experimenter received instructions about the type of touch that needed to be administered during the trial. These instructions also indicated which of the two marked areas on the participant’s forearm needed to be touched. The instructions were followed by a 6 second countdown, after which the trial started. Trials were 8 seconds in duration. While the touch stimulus was immediately administered at the start of a trial, noise clicks were only played through the in-ear earphones after 2 seconds. This delay was used to ensure that the PAR would be evoked during the actual experience of the touch stimulus. As can be seen in Fig 2, trials were always presented in blocks of two. The same tactile stimulus was presented in trials of a corresponding block. After each block, participants filled out the VAS to indicate the perceived pleasantness of the touch. There were 4 blocks and 8 trials per touch condition. An additional 16 trials (8 blocks) were used to record a baseline for the PAR. These trials were 6 seconds in duration. During these trials, the participants only heard the noise clicks that were used to evoke the PAR. No other stimuli were presented and participants were instructed to simply look at the fixation cross. Participants did therefore also not have to fill out the VAS after these baseline trials. The order of all blocks was randomized for each participant. An additional restriction was added to this randomization to ensure that, within a series of 12 blocks, the four touch conditions were presented twice (once on each of the two marked areas on the forearm). There was always one baseline condition presented within a sequence of 3 blocks, meaning that 4 baseline conditions were presented in a series of 12 blocks. This randomization ensured that the same touch condition was never repeated more than twice a row within a series, which reduced the chance of repetition effects occurring [38]. At the end of the main experiment, participants filled out the LFT VAS and indicated how similar they perceived the social and robotic touch to be.

Participants subsequently completed the control experiment, which was designed to validate the PAR measurements and replicate the reflex’s specific sensitivity to primary rewards. In this experiment, the PAR was measured while participants viewed pleasant (adventure, erotic, food, nurturant), neutral (buildings, humans, landscapes, objects) and negative (disgust, mutilation, threat, victim) images and while they viewed messages communicating that the participant earned or lost (0.5 and 1 euro per trial). A full description of the control experiment can be found in S1 File. Participants received either credits (1.5 PPU) or money (9 euro), in addition to the earnings from the control experiment (3 euro), as compensation. The total experiment took around 90 minutes to complete. The study was approved by and executed in according with the regulations of the ethical board of the Faculty of Social and Behavioural Sciences, Utrecht University (24–0230).

### 2.6. Design and data-analysis

JASP (Version 0.18.3.0) was used for the data-analysis. A 2x2 mixed subjects design was used, with velocity (CT-optimal vs. CT non-optimal touch) and mode (social vs. robot touch) as within-subjects factors and LFT as between-subjects covariate. The average LFT score was calculated across all data-points, which was subsequently used to mean-center the covariate. Applying the criteria described in a previous section (see *physiological recordings and preprocessing*) increased the number of missing values in the dataset, as we were only able to use the data from one ear for twelve participants. A linear mixed model was therefore used for the primary analysis, due to its superior ability to deal with missing data (see, e.g., [39]). A total of 43 participants and 546 observations were used for the analysis. Recording site (ear) and participant number were used as random effects, with velocity, mode (social vs. non-social touch), subjective rating and LFT as fixed effects. A second linear mixed model was used to assess differences in the subjective ratings, with participant number as random factor and velocity, mode and LFT as fixed factors. Assumptions of linearity, normality and homoscedasticity were checked and met. For all analyses α = 0.05 (two-tailed).

## 3. Results

### 3.1. PAR

**Fig 3:**
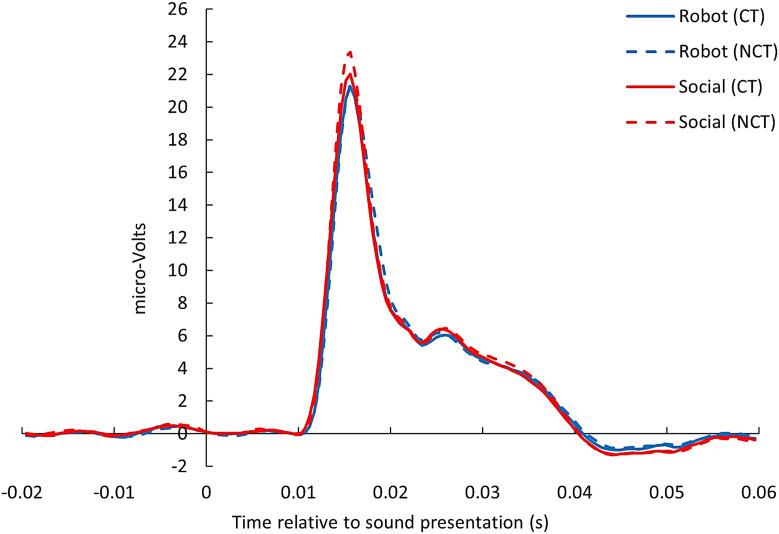
Post-auricular muscle activity (averaged across ears). Averaged post-auricular muscle activity time-locked with sound presentation (x = 0) and baseline-corrected for activity during the preceding 50ms. CT = CT-optimal touch. NCT = CT non-optimal touch.

**Fig 4:**
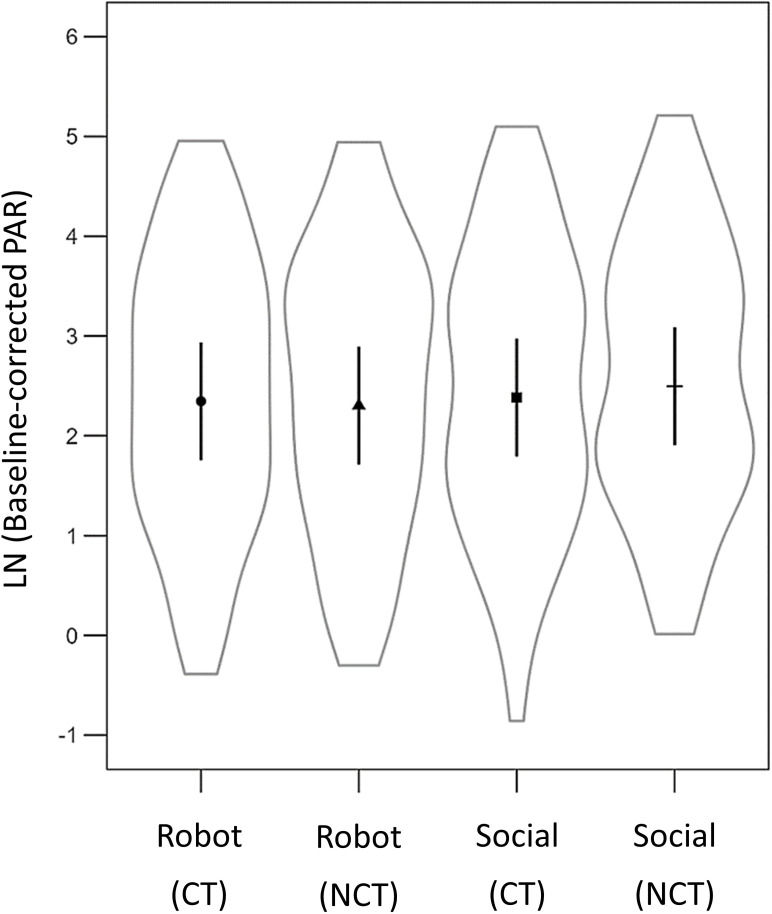
Average baseline-corrected PAR for the four touch conditions. CT = CT-optimal touch. NCT = CT non-optimal touch. Lines represent a 95% confidence interval.

Fig 3 shows the raw (not-baseline-corrected) averaged post-auricular muscle activity for each touch condition, illustrating the reliability of the PAR measurements. The regression coefficients of the linear mixed model of your primary analysis are displayed in Table 1. In contrast to our hypotheses, there was no significant main effect for velocity (*EMM*_*CT-optima*l _= 2.37, *SE*_*CT-optimal*_ = 0.24; *EMM_CT non-optimal _*= 2.40, *SE_CT non-optimal_* = 0.24) or LFT (see Table 1; Fig 4). However, there was a significant difference between social touch and robotic touch. As expected, a significantly stronger potentiation was observed for social touch (*EMM* = 2.44, *SE* = 0.24) than for robotic touch (*M* = 2.33, *SE* = 0.24).

**Table 1:** Regression coefficients with the LN(PAR) as outcome.

	Coefficient	SE	t	*p*
Intercept	2.44	0.31	7.95	< .001
LFT	0.01	0.01	0.63	.534
Subjective rating	−1.05 × 10^−4^	3.41 × 10^−4^	−0.31	.759
Velocity	−0.02	0.03	−0.66	.507
Mode	−0.06	0.03	−2.20	.028
Mode * Velocity	0.04	0.03	1.50	.133

### 3.2. Subjective ratings

**Fig 5:**
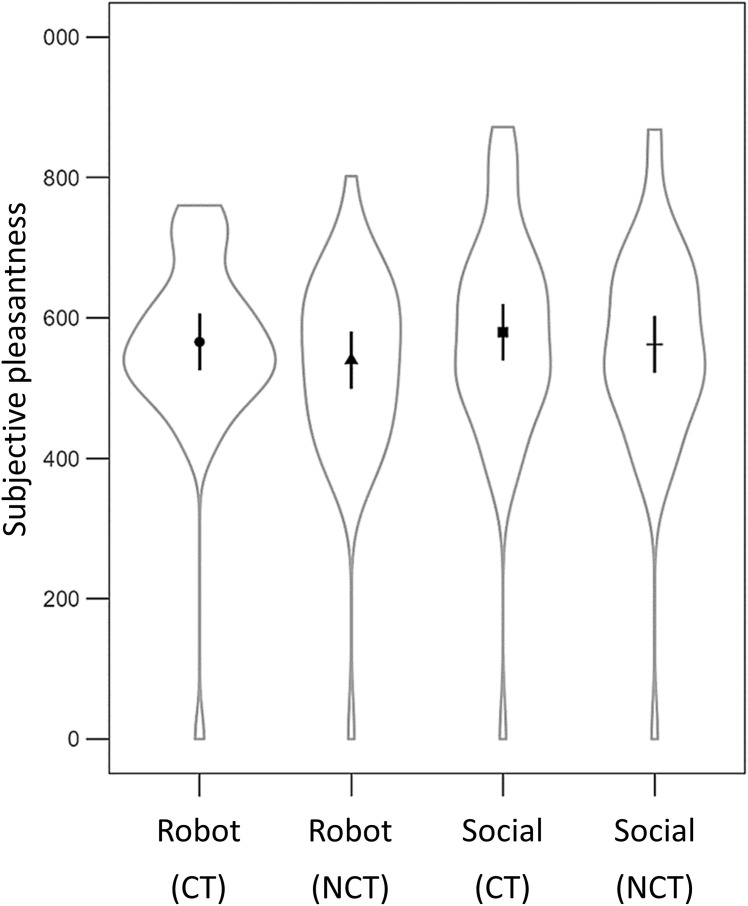
Average subjective pleasantness for the four touch conditions. CT = CT-optimal touch. NCT = CT non-optimal touch. Lines represent a 95% confidence interval.

Significant effects were found for both the velocity and mode of touch (see Table 2; Fig 5). Consistent with our expectations, CT-optimal touch was rated as significantly more pleasant (*EMM* = 572.75, *SE* = 19.51) than non-CT optimal touch (*EMM* = 551.06, *SE* = 19.53). In addition, social touch was rated as more pleasant (*EMM* = 571.01, *SE* = 19.50) than robotic touch (*EMM* = 552.80, *SE* = 19.55). There was no significant main effect for LFT, which is in contrast to our hypothesis.

**Table 2:** Regression coefficients with the subjective pleasantness as outcome.

	Coefficient	SE	t	*p*
Intercept	561.49	19.25	29.17	<.001
LFT	1.14	1.21	0.94	.351
Velocity	10.85	3.21	3.38	<.001
Mode	−9.11	3.23	−2.82	.005
Velocity * Mode	2.17	3.21	0.68	.499

### 3.3. Control experiment

Full analyses and results for the control experiment are reported in the supplemental materials. In short, the PAR was potentiated more during the viewing of positive images (*EMM* = 1.52, *SE* = 0.22) compared to negative (*EMM* = 1.33, *SE* = 0.22) and neutral (*EMM* = 1.38, *SE* = 0.22) images. Within the positive images category, images of food (*EMM* = 1.60, *SE* = 0.22) and erotica (*EMM* = 1.70, *SE* = 0.22) potentiated the PAR significantly more than the adventure (*EMM* = 1.37, *SE* = 0.22) and nurturant (*EMM* = 1.37, *SE* = 0.22) images. Modulation of the PAR was not different for messages communicating monetary rewards (high reward, *EMM* = 1.51, *SE* = 0.23; low reward, *EMM* = 1.47, *SE* = 0.23) and monetary losses (high loss, *EMM* = 1.51, *SE* = 0.23; low loss, *EMM* = 1.69, *SE* = 0.23). Taken together, these results are in line with our expectations and confirm that the PAR is particularly responsive to primary and not secondary rewards.

## 4. Discussion

The aim of the current study was to investigate if social and CT-optimal touch could modulate the PAR, and assess if the reflex could be used as an implicit measure of pleasantness perception. We hypothesized that CT-optimal touch would be subjectively perceived as more pleasant than CT non-optimal touch, and that it would additionally result in a stronger potentiation of the PAR. Based on the nursing hypothesis [25], we also expected that potentiation would be stronger for social touch than for robotic touch. In all analyses, we controlled for the influence of LFT and expected this to be positively associated with both the subjective pleasantness perception and modulation of the PAR. The results are partially in line with these hypotheses. While CT-optimal touch was indeed subjectively perceived to be more pleasant than CT non-optimal touch, it was not associated with a stronger modulation of the PAR. However, potentiation of the PAR was significantly stronger for social touch than for robotic touch. Social touch was also subjectively perceived to be more pleasant than robotic touch. Despite these parallel observations, subjective pleasantness ratings were not significantly associated with modulation of the PAR. In contrast with our hypotheses, LFT was not significantly related to either the subjective ratings or modulation of the PAR. The results of our control experiments are in line with previous observations [20], as they confirmed that the PAR is modulated more by primary (e.g., appetitive-related images) than secondary rewards (e.g., non-appetitive-related images and monetary rewards).

It is unexpected that CT-optimal touch did not induce a stronger potentiation of the PAR than CT non-optimal touch. We based our initial hypothesis on the observations that the modulation of the PAR is stronger for pleasant stimuli [18,21–23], and that CT-optimal touch tends to be subjectively perceived as more pleasant than CT non-optimal touch [9]. While both were indeed observed in the current study, potentiation of the PAR was not significantly different for CT-optimal and CT non-optimal touch. It should also be noted that no significant association between the subjective pleasantness ratings and modulation of the PAR. Based on these results, it cannot be concluded that the PAR can be used to implicitly assess the pleasantness perception of CT-optimal touch. Since this is the first study to assess how the PAR responds to tactile stimuli, there is limited literature that can be used to interpret the current results. A partial explanation could potentially be derived from the nursing hypothesis, as this states that the reflex is modulated most by stimuli that are closely associated with nursing [25]. Although we initially assumed that this association would be strongest for CT-optimal touch [27], the insignificant results in the current study could suggest that nursing is not strongly associated with a specific touch velocity. As neither velocity was perceived to be unpleasant, it is possible that nursing is instead more broadly associated with pleasant forms of touch. It is more difficult to explain why these insignificant physiological results were observed alongside a significant subjective preference for CT-optimal touch. The PAR is thought to reflect positive emotional [24] and appetitive motivational [20] processes. An association between potentiation of the reflex and the subjectively perceived pleasantness of touch could therefore have been expected. Previous studies have not investigated the relation between PAR modulation and subjective valence ratings (see, e.g., [18,20,23]), which further complicates the interpretation of the current results. Additional research is therefore needed to understand to what extent the PAR actually corresponds to subjective measures, and which factors can cause a discrepancy between them.

In line with our expectations, our results do demonstrate that the PAR can be differentially modulated by social and robotic touch. This finding is compatible with the aforementioned nursing hypothesis [25], which argues that nursing-related stimuli would have become associated with the PAR through evolutionary processes. This has already been observed for appetitive stimuli such as food or images of breasts [20]. Since interpersonal touch plays an important role in the nursing process [26], it can be expected that this would have become similarly associated with the PAR. The reason that this association is unlikely to extend to robotic touch is because social touch encompasses more than mere tactile stimulation. It is not only used to convey emotions [40] but also signals the very presence of another human being. This could especially be important in the context of nursing, as this means that the provider of the food is nearby. Social touch is therefore expected to have more appetitive value and be more closely associated with nursing, which would subsequently be reflected in a stronger modulation of the PAR. This hypothesis is further supported by two other observations. First, social touch was also subjectively perceived to be more pleasant than robotic touch in the current study. While this supports the appetitive value of social touch, it should again be noted that these subjective ratings were not directly associated with PAR potentiation. Second, we found that the PAR particularly potentiates during the perception of primary rewards. This underscores that the increased modulation in response to social touch might indeed reflect nursing-related appetitive value.

The current results do, however, contrast with studies in which no perceptual differences between human and robotic touch were found (see, e.g., [28,29]). This has not only been observed for subjective pleasantness ratings, but also for associated EEG patterns [29]. There are several possible explanations for these conflicting results. First, it could be related to the perceived humanness of the robotic touch. Materials that feel more human-like are perceived to be more pleasant [41]. In the current study, participants were asked to retrospectively reflect on the similarity of the different types of touch. The average rating was relatively low, indicating that the social and robot touch were not perceived to be very similar. Different robotic devices and materials were used in studies by Triscoli et al. (2013) [28] and Zheng et al. (2021) [29]. It is possible that these were perceived to be more human-like, which would explain why they did not observe significant differences in their studies. Second, the visibility of the robot could have had an influence. Although participants were instructed to look at a fixation cross during the experiment, they were able to see the robot when they came into the lab. This differs from previous studies, as participants either closed their eyes [29] or were wearing glasses that blocked their peripheral vision [28]. Triscoli et al. (2013) did find that the perceived pleasantness of touch was not altered when participants were aware that they were being touched by a robot [28]. However, their participants never actually saw the robot during the experiment. Being able to see the robot, as was the case in our experiment, might emphasize the inhumanness of the device and subsequent touch, therefore leading to a reduced perceived pleasantness. This seems compatible with the observation that videos of robotic touch are perceived to be less pleasant than videos of human touch [42]. It would be interesting to further explore the potential influence of visual cues on the pleasantness perception of robotic touch in future studies.

Contrary to our expectations, LFT was not significantly associated with either the PAR or the subjective ratings. Based on Meijer et al. (2022), we hypothesized that LFT would be positively associated with the pleasantness perception of CT-optimal and CT non-optimal touch and the subsequent modulation of the PAR [32]. Although our findings are in contrast with this hypothesis, they are compatible with the results from other studies. For example, Hebert et al. (2015) found that the PAR was only modulated when participants actively viewed images of food, but not when they were anticipating them [43]. They took this as an indication that the reflex might be more indicative of hedonic liking rather than motivational wanting. Similar results have been observed by Mercado (2014) [44]. It is then perhaps less likely that LFT is directly associated with modulation of the PAR, given that it reflects the current wanting of social touch.

It is, however, still surprising that no relation between LFT and the subjective ratings of CT-optimal and CT non-optimal touch was found. A potential explanation is that the current study focused on touch that was administered by a stranger and a robotic device. In contrast, Meijer et al. (2022) used videos to measure the vicarious perception of CT-optimal and CT non-optimal touch. These videos depicted a hand stroking the forearm of another person [32]. Although the vicarious and physical perception of touch elicit a similar neural response [45], an important difference is that set-up of the videos allow participants to imagine the social setting in which the tactile interaction occurs. This is relevant because the deprivation of a need, such as LFT, is thought to only be associated with the enhanced perceptual appeal of need-relevant stimuli (i.e., stimuli that can successfully satiate the deprived need; [46,47]). Touch from a stranger is less desired [31] and perceived as more unpleasant [48] than touch from close social connections, and may subsequently be less need-relevant. Participants in the study by Meijer et al. (2022) could have imagined that touch occurred in a close social relationship [32], thereby increasing the perceived need-relevance of the tactile stimuli and consequently making it easier to find a significant association with LFT. While this can naturally not be concluded based on the available data, it would be interesting to consider a potential modulatory influence of relationship closeness on the association between LFT and the pleasantness perception of touch in future research.

The current design also has three important limitations that need to be addressed. First, we did not include an unpleasant form of touch as a control condition. Such stimuli could provide additional insights into how the PAR is modulated by different types of touch. Previous research has shown that unpleasant images inhibit the PAR (see, e.g., [18]). It would be interesting to investigate if the same can be observed for unpleasant touch. In the current study, CT non-optimal was used as a less pleasant form of touch. However, as previously mentioned, while subjective ratings were indeed significantly lower for CT non-optimal touch, participants did generally not seem to perceive it as unpleasant. Future studies should therefore aim to incorporate an actual unpleasant, but non-painful, form of touch. One possibility is to use a rough material to administer touch [49,50], as this is perceived to be significantly more unpleasant than the use of soft materials [50]. Second, we did not regulate the temperature and pressure of social touch in the current study. As both are associated with the activation of CT-fibers [51,52], variability in temperature and pressure could have affected how social touch was perceived. We can therefore also not exclude the possibility that this influenced the observed physiological and subjective differences between social and robotic touch. It is therefore important to regulate temperature and pressure in future studies. Thermal consistency could be ensured by using cotton gloves [53] or by making the experimenters rub their hands together before the experiment [54,55]. Regulating pressure might be more difficult for skin-to-skin contact, but the marked areas on the forearm could be narrowed so that the contact area and splay of the finger can be more easily monitored. Third, we randomized block order and restricted the number of times a participant could receive the same touch condition consecutively. This was done following the observation that repeated exposure can affect the subjective pleasantness perception of touch [38]. While we wanted to minimize the potential influence of these repetition effects in the current study, it could actually be interesting to investigate if repeated exposure similarly affects potentiation of the PAR in future research. This can be achieved by removing randomization restrictions and controlling more directly for block order in future analyses.

To conclude, our results are the first to demonstrate that the modulation of the PAR is stronger for social touch than for robotic touch. This increased potentiation is seems to reflect the primary reward value of social touch. These results do not only provide valuable new insights into the PAR, but also into the general physiological response to social touch. Although the current results do not suggest that the PAR can be used to implicitly assess the pleasantness perception of CT-optimal touch, it is important to consider the methodological limitations of the current design and the absence of comparable studies. Additional research is therefore needed before conclusions can be drawn about the full usability of the PAR as an implicit measure of tactile pleasantness. Future studies should, among others, aim to include an unpleasant type of touch and further regulate the application of social touch. These studies are needed to better understand the modulation of the PAR and how this relates to the pleasantness perception of touch.

## Supporting information

S1 File Control experiment. (DOCX)

S2 File Data and analyses. (ZIP)
