# Supplementary material for: Modulation of the post-auricular reflex in response to social and CT-optimal touch
Source: PLoS One. 2025 Aug 13;20(8):e0329625. doi: 10.1371/journal.pone.0329625 (PMC12349074; doi:10.1371/journal.pone.0329625)
Supplement: S1 File — (DOCX) [file pone.0329625.s001.docx]

Supplementary materials to:

**Modulation of the post-auricular reflex in response to social and CT-optimal touch**

B. Hasenack^1,2*^, A. Keizer^1^, O. Wódecka^1^, L. B. Schaafsma^1^, H. C. Dijkerman^1^ & D. Terburg^1,3^

^1^ Faculty of Social and Behavioural Sciences, Experimental Psychology, Utrecht University, Utrecht, The Netherlands.  ^2^ Faculty of Social and Behavioural Sciences, Clinical Psychology, Utrecht University, Utrecht, The Netherlands.
^3^ Department of Psychiatry and Mental Health, University of Cape Town, Cape Town, South Africa.

^*^ Corresponding author | [b.hasenack@uu.nl](mailto:b.hasenack@uu.nl) | Heidelberglaan 1, 3584 CS, Utrecht.

**Methods control experiment**

*Procedure*

The control experiment was included in this study to validate our PAR measurements and replicate previous findings on PAR-modulation by primary and secondary rewards. The experiment was similar to the visual part of the experiment reported by Benning (2011) with added trials to assess the PAR’s sensitivity to monetary rewards and losses.

Each trial followed the same order: Fixation (1s), announcement (3s), fixation (2s), stimulus (~6s). Fixation screens consisted of a black ‘+’ on a gray background and participants were instructed to fixate their gaze on this fixation-cross. The announcement screen announced the type of trial, either ‘This trial you will see an image’ or ‘This trial you might win or lose money’. Click sounds were delivered simultaneously with the stimulus screen in the same fashion as in the touch experiment (i.e. 60 clicks with a rate of 10 Hz and a jitter of 10 ms). The stimulus screen presented either an affective image or a monetary outcome.

Affective images were taken from the International Affective Picture System (IAPS; Center for the Study of Emotion and Attention, 1999) database based on the study of Benning (2011). These included images with negative, neutral and positive valence. The sixteen negative images were selected from the IAPS-categories: disgust (IAPS-codes: 9342, 9520, 9560, 9830), mutilation (3051, 3061, 9253, 9420), threat (6250, 6260, 9630, 6190) and victim (6570, 6312, 6540, 6561). The sixteen neutral images were selected from the categories: buildings (5731, 7180, 7490, 7491), humans (2190, 2393, 2870, 2890), landscapes (5120, 5390, 5740, 9210) and objects (7002, 7004, 7034, 7038). The seventeen positive images were selected from the categories: adventure (5623, 8034, 8180, 8210), erotic (4640, 4660, 4680, 4255, 4572), food (7200, 7230, 7260, 7460) and nurturant (2071, 2160, 2340, 2655). The erotic condition contained five instead of four images because image-4255 was specifically included for participants attracted to women and image-4572 for participants attracted to men.

The positive and negative images were matched for arousal. The mean valence and arousal ratings for the three main conditions can be found in Table 1. Independent t-tests showed that: positive compared to neutral images are scored higher in valence (*t*(31)=14.3, *p*<.001) and arousal (*t*(31)=14.5, *p*<.001); negative compared to neutral images are scored lower in valence (*t*(31)=-18.1, *p*<.001) and higher in arousal (*t*(31)=14.8, *p*<.001); positive compared to negative images are scored higher in valence (*t*(31)=28.1, *p*<.001), but similar in arousal (*t*(31)=1.3, *p*=.218).

**Table 1**

*Mean valence and arousal ratings*

|  | **Mean valence (range)** | **Mean arousal (range)** |
| --- | --- | --- |
| Positive images | 7.20 (6.06-8.03) | 5.46 (4.57-6.59) |
| Neutral images | 5.00 (4.39- 5.59) | 2.82 (2.00-3.43) |
| Negative images | 2.55 (2.00- 3.57) | 5.75 (4.49-6.93) |

The monetary outcome screen communicated either a gain (low or high: 0.5 or 1 euro), a loss (low or high: 0.5 or 1 euro) or an even outcome (no loss or gain). These were communicated with the messages: ‘you win: +0.50 (or +1.00) euro’, ‘you lose: -0.50 (or -1.00) euro’ and ‘you play even: +/- 0 euro’. Six even-outcome, high-gain and low-gain trials, as well as four high-loss and low-loss trials, were included ensuring that each participant gained 3 euro in total.

Combined this resulted in a total of 75 trials which were presented in random order. The experiment started with two practice trials (a monetary reward trial with a ‘you play even’ outcome and a random neutral image) to get acquainted with the procedure.

*Analysis*

PAR data was pre-processed in the same manner as in the touch experiment. As the errors in touch application and missing questionnaire data reported as exclusion criteria in the main manuscript was not an issue for the control experiment data, statistical analysis could be done on data from 47 participants. Three linear-mixed-models (LMMs) were estimated. Firstly, a model to assess the effect of image-valence on the PAR with valence (negative, neutral and positive) as fixed factor. Secondly, a model to assess the effect of the different categories within the positive images with category (adventure, erotic, food and nurturant) as fixed factor. Thirdly, a model to assess the effect of monetary reward with reward-type (loss, gain) and reward-magnitude (0.50 euro, 1.00 euro) as fixed factors. All three models contained participant and ear as random factors and from the erotic images only PAR data was included from the image that aligned with the participant’s sexual preference.

**Results control experiment**

In the first model, the overall effect of image-valence on the log-transformed PAR was significant (*F*(2, 898.20)=5.452, *p*=.004), which was due to a significantly stronger PAR during positive (*EMM*=1.52, *SE*=0.22) compared to negative (*EMM*=1.33, *SE*=0.22; *Z*=3.159, *p*=.002) and neutral (*EMM*=1.38, *SE*=0.22; *Z*=2.407, *p*=.016) image-presentation. PAR was not different for negative and neutral image-presentation (*p*=.449). Thus, the PAR increased when viewing positive compared to neutral and negative images.

In the second model, the overall effect of image-category within the positive images on the log-transformed PAR was also significant (*F*(3, 267.42)=4.710, *p*=.003), which was due to a significantly stronger PAR during erotic (*EMM*=1.70, *SE*=0.22) compared to adventure (*EMM*=1.37, *SE*=0.22; *Z*=3.030, *p*=.002) and nurturant (*EMM*=1.37, *SE*=0.22; *Z*=3.033, *p*=.002) image-presentation, as well as a significantly stronger PAR during food (*EMM*=1.60, *SE*=0.22) compared to adventure (*Z*=2.124, *p*=.034) and nurturant (*Z*=2.127, *p*=.033) image-presentation. PAR was not different when comparing erotic and food image-presentation (*p*=.378) or when comparing adventure and nurturant image-presentation (*p*=.998). Thus, the PAR increased when viewing food and erotic, compared to adventure and nurturant, images.

In the third model the main effects of reward-type (*F*(1, 263.72)=2.335, *p*=.128), reward-magnitude (*F*(1, 263.72)=0.926, *p*=.337) as well as their interaction (*F*(1, 263.72)=2.061, *p*=.152) were not significant. Thus, monetary rewards and losses did not modulate the PAR.

**References**

Benning, S. D. (2011). Postauricular and superior auricular reflex modulation during emotional pictures and sounds. *Psychophysiology*, *48*(3), 410–414. <https://doi.org/10.1111/j.1469-8986.2010.01071.x>
